# Neighborhood disorder impacts cognitive processing during navigation on a novel virtual reality paradigm

**DOI:** 10.1038/s41598-026-56852-4

**Published:** 2026-06-09

**Authors:** May I. Conley, Nick Townsend, Arielle Baskin-Sommers

**Affiliations:** 1 Department of Psychology, Yale University, 100 College St., New Haven, CT 06510 USA; 2 Child Study Center, Department of Child and Adolescent Psychiatry, NYU Langone Medical Center, New York, NY 10016 USA

**Keywords:** Neighborhood disorder, Cognitive processing, Wayfinding, Risky behavior, Ecological validity, Neuroscience, Psychology, Psychology

## Abstract

Living in neighborhoods characterized by physical and social disorder (e.g., litter, abandoned buildings, crime) is associated with poorer cognitive functioning. However, much of this work relies on decontextualized laboratory tasks, limiting insight into how everyday environmental experiences shape day-to-day cognitive functioning. Here, we introduce the Neighborhood Errand Task (NET), a novel navigation paradigm designed to approximate memory and information processing during wayfinding within disordered neighborhood contexts. Participants first learned a route through map study and guided practice, then completed navigation trials requiring choices between learned-familiar routes and novel-shortcut routes. Neighborhood disorder was experimentally manipulated via environmental cues and intermittent surprise “robbery” events. A diverse sample of U.S. adults (N = 100) completed the NET alongside self-reports of perceived neighborhood disorder and risky/impulsive behavior. Greater perceived neighborhood disorder was associated with poorer wayfinding in the learned route and shortcut trials, which may reflect differences in information processing, caution, strategy selection, or specific memory processes. Furthermore, inefficient navigation amplified the relationship between perceived neighborhood disorder and engagement in risky/impulsive behavior, suggesting that cognitive inefficiencies and environmental perceptions heighten vulnerability to behavioral dysregulation. By embedding cognition in a realistic, navigable context, the NET highlights how environmental adversity may shape adaptations relevant to survival.

**Supplementary Information:**

The online version contains supplementary material available at 10.1038/s41598-026-56852-4.

## Introduction

For many individuals, daily life involves navigating contexts rife with unpredictability, disorder, and diminished safety. In fact, 40% of U.S. adults report worrying about their personal safety in their neighborhoods^1^. A growing body of research suggests that residing in a neighborhood with heightened physical and social disorder (e.g., excessive litter, abandoned buildings, crime and violence) is associated with difficulty across a range of cognitive functions^2–6^. Importantly, it is not simply objective neighborhood conditions that matter. Individuals living in the same objectively disordered environment may differ in how much disorder they perceive, and these individual differences in perceived disorder are independently associated with worse cognitive functioning even after accounting for objective neighborhood characteristics^7–9^. Indeed, subjective perceptions of neighborhood disorder have been shown to more consistently predict cognitive performance than objective indicators, with perceived disorder linked to poorer episodic memory, working memory, executive function, and spatial ability. These findings have led to proposals that cognitive functions may adapt to promote survival in environmental contexts with unpredictable adversity including neighborhood disorder^10,11^. However, much research on neighborhoods and cognition relies on tightly controlled, decontextualized laboratory tasks^12^ that isolate specific components of cognition and may not capture how cognition operates during forms of real-world behavior that are essential for survival in unpredictable environments.

One everyday behavior that is critical for survival is wayfinding—the process of navigating to goal locations in an environment^13–15^. Importantly, successful wayfinding in everyday life reflects the coordination of multiple cognitive processes that interact with dynamic information in individuals’ environments^16,17^. For example, day-to-day wayfinding requires encoding and retrieving spatial and contextual information, updating representations of the environment as new information is acquired, planning routes, and attending to and flexibly responding to relevant cues^13,15,18^. Wayfinding performance also is related to individual differences in strategy selection, such as relying on learned routes or taking shortcuts or incorporating other heuristics during decision-making^19,20^. Individual differences in wayfinding strategies have been related to brain activation, with greater striatal activation associated with habit-based learned route responding and greater hippocampal activation associated with flexible shortcut use. Notably, these processes can be modulated by environmental demands, such that stressful or unpredictable conditions may bias individuals towards habit-based, less efficient and less flexible responding during wayfinding^21–23^ potentially due to the role of stress in modulating hippocampal- versus striatal-based processing^24^. Taken together, wayfinding provides a window into how multiple cognitive processes interact to support goal-directed behavior in real-world environments.

In experimental settings, wayfinding is often evaluated using the dual-solution paradigm (DSP), which involves learning the locations of arbitrary objects–like a wishing well or lion statue–in a virtual, abstract maze made of brick walls and subsequently navigating to each location following a “learned route” or “shortcut”^25^. Research using the DSP suggests that while some individuals have a greater tendency for following the learned route (indexing habit-based responding) or taking shortcuts (indexing flexible retrieval), many people use a mixture of these strategies during wayfinding. While the DSP has led to valuable insights about navigation strategies during wayfinding, it stands to reason that navigating to a wishing well is quite different from the wayfinding challenges people face in everyday life, such as in the face of neighborhood disorder.

Differences in performance on abstract maze paradigms and wayfinding in the real world are important to highlight, as the cognitive processes thought to support wayfinding are also implicated in the regulation of behavior more broadly. For example, deficits in memory encoding and retrieval, core components of both navigation and adaptive information processing, have been associated with a range of risky and impulsive behaviors^26^ including problematic substance use^27,28^, risky sexual behavior^29^, and disinhibited eating^30^. Similarly, the attentional and executive functioning processes that allow individuals to orient toward goals, inhibit competing responses, and flexibly update plans during navigation are also frequently disrupted among those who engage in higher rates of risky and impulsive behaviors^31,32^. Nevertheless, existing studies share a critical limitation in that they assess cognitive processes in isolation rather than capturing the dynamic interdependence among processes that characterizes goal-directed behavior in real-world environments^33^.

To move beyond abstract maze paradigms, researchers have increasingly examined wayfinding and navigation-related processing using virtual reality-based tasks that attempt to simulate the real-world settings individuals interact with in their day-to-day lives. Many of these tasks incorporate relatively structured and familiar everyday settings such as virtual supermarkets^34^, indoor hallway networks^35^, or simplified low-density residential areas (i.e., suburbs)^36^ to examine spatial learning and navigation performance. More complex virtual environments also have been developed to approximate navigation through larger more spatially detailed settings such as towns^21^, college campuses^37,38^, and forest-like environments^39^. These paradigms are considered to have enhanced ecological validity by increasing the representativeness (i.e., how well a phenomenon can be studied in a manner that resembles the real-world) of task-based navigation environments while preserving experimental control^12,40,41^. However, they vary in the specific components of navigation they assess–ranging from route planning between landmarks with lower representativeness such as celebrity faces or pieces of fruit to directional pointing–making it difficult to generalize findings across tasks or capture how navigation unfolds during goal-directed behavior in more realistic settings. These design choices matter because experimental control is often achieved at the cost of ecological fidelity. Truly understanding the interplay between environmental context and cognition requires navigation paradigms in which both the task context and motivating goals closely mirror those encountered by the target population in their everyday lives^42–44^.

To address the limitations of existing research on neighborhood, cognition, and wayfinding tasks—and to link cognitive functions to individuals’ environmental contexts and real-world behavior—we developed the Neighborhood Errand Task (NET). The NET is a novel virtual navigation paradigm designed to embed wayfinding within a neighborhood context with ecologically-relevant indicators of physical and social disorder. Our goal was to model the real world as it is experienced by people who live in disordered neighborhoods and examine how individual differences in that lived experience impact wayfinding and risky/impulsive behavior. Adults recruited from a region of the U.S. characterized by elevated crime rates and variability in crime concentration completed the NET along with self-report measures of neighborhood disorder and risky and impulsive behavior. Following from previous research, our approach is grounded in the premise that neighborhood disorder does not affect everyone equally; even among people living in the same high-crime area, individuals differ in how much disorder they perceive, and it is these differences in subjective experience that may shape cognitive and behavioral outcomes. We hypothesized that adults who reported higher perceived neighborhood disorder would show poorer wayfinding performance during the NET. Further, we expected that adults with greater difficulty in wayfinding would engage in more real-world risky and impulsive behavior. By embedding a cognitive task within a realistic virtual context, this study aims to advance ecologically representative approaches to studying cognitive processing in everyday environments and provide insights into vulnerability to behavioral dysregulation.

## Materials and methods

### Participants

Adults were recruited from New Haven County, Connecticut through flyers soliciting risk-taking behavior or asking if people have been exposed to violence. New Haven ranks in the 97th percentile for crime, with an average of 309 crimes per square mile compared to the national median of 24.5 (Data accessed from http://www.neighborhoodscout.com/ct/new-haven/crime/new-haven/crime/ on 04/06/2026). This, combined with our unselected community sample recruitment strategy, yielded a sample with meaningful variability in exposure to, and perceptions of, neighborhood disorder and engagement in risky behavior.

A phone screen was used to exclude individuals who were younger than 18 or older than 65; were diagnosed with schizophrenia, bipolar disorder, or psychosis, not otherwise specified or had a family history of psychosis; took antipsychotic, anticonvulsant, or mood stabilizers; or had medical problems that could impede comprehension of or performance on the task. Individuals who passed the phone screen were invited to the lab. Prior to completing in-person activities, each participant received a detailed explanation of all study procedures including potential risks and benefits, as well as their right to decline or discontinue participation at any time without penalty. All participants provided written informed consent in compliance with the guidelines established by the Yale University Human Subjects Committee and all research was performed in accordance with the Declaration of Helsinki.Following consent, each in-person session began with screening to exclude participants who scored below the fourth-grade level on a standardized reading measure or below 80 on a standardized IQ measure, as performance below these thresholds could indicate difficulty comprehending study materials. Participants earned $15/hour for their participation. Supplemental Table 1 provides summaries of participant characteristics for the final sample (*n* = 100).

## Measures

### Perceived Neighborhood Disorder (PND) Scale

The PND scale is a 15-item self-report measure of physical (e.g., vandalism, abandoned buildings, cleanliness) and social (e.g., crime, loitering) disorder in one’s neighborhood. Items are rated using a 4-point Likert-type scale (1 = “Strongly Disagree” to 4 = “Strongly Agree”)^45^. Scores were summed into a total PND score (range 15–60) with higher scores indicating greater PND (Supplemental Fig. 1 for a histogram of PND scores in the present sample).

**Fig.1:**
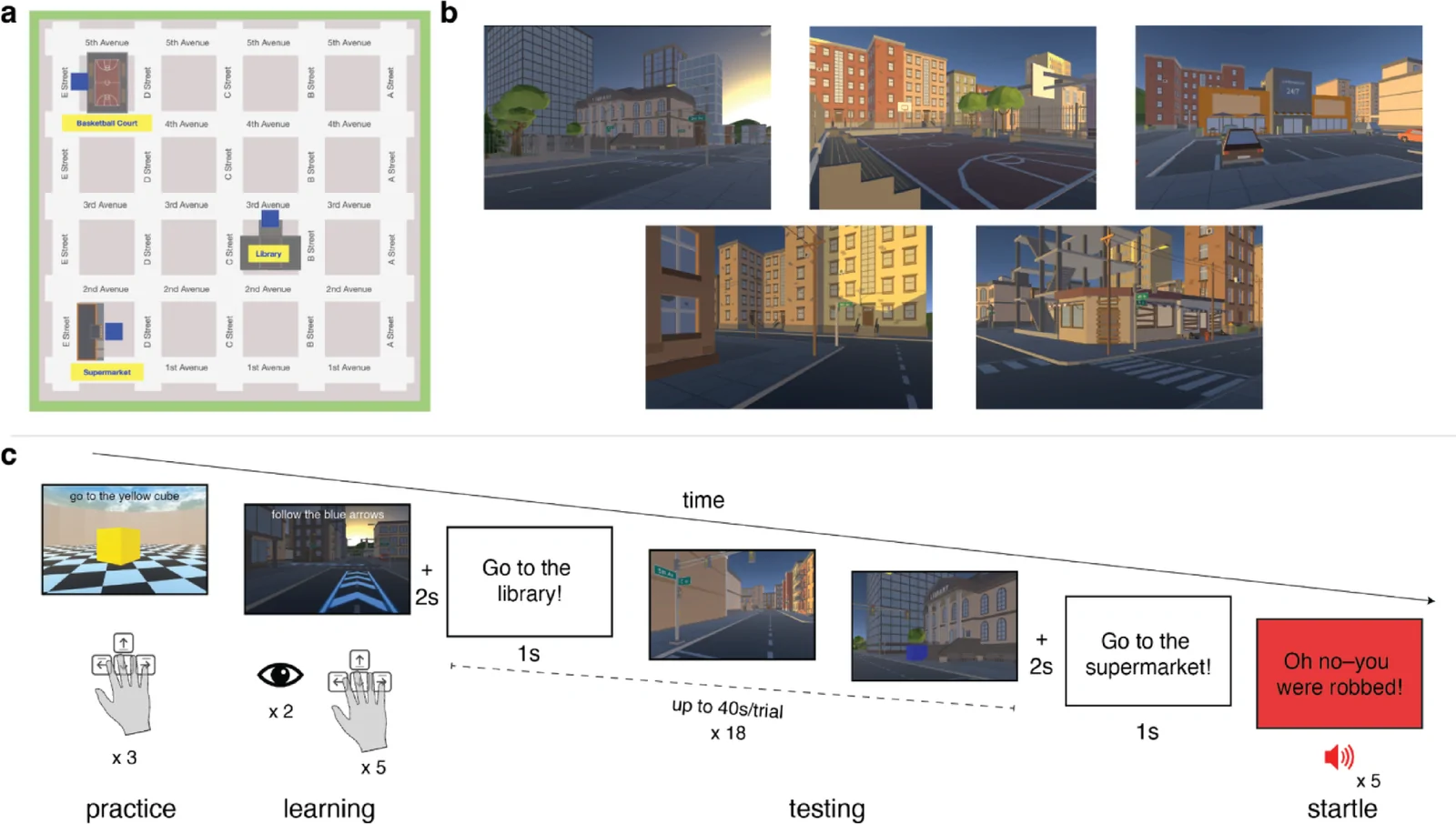
Task Overview. (**a**) During learning, participants were given 2 min to review a simplified overhead map showing goal locations in the virtual neighborhood; (**b**) participants’ view of various locations including (in clockwise order) the library, basketball court, supermarket, and side streets; (**c**) schematic of the overall task procedure. First, participants were given the opportunity to practice walking around in a practice virtual environment. Next, participants were guided by the experimenter and practiced navigating themselves along the learned route. Following learning, testing included 18 randomly ordered trials with 5 surprise startle trials interspersed to increase the sense of discomfort, unease, and uncertainty that characterizes disordered environments in the virtual neighborhood.

## Risky, Impulsive, and Self-Destructive Behavior Questionnaire (RISQ)

The RISQ is a 38-item self-report measure of risky, impulsive, and self-destructive behaviors across 8 domains (aggression, self-harm, gambling, impulsive spending/driving, impulsive eating, risky sex, illegal behavior, and alcohol use)^46^. Lifetime frequencies were summed to generate a total score, with higher scores indicating greater lifetime engagement in risky, impulsive, and self-destructive behaviors.

## Tasks

### NIH Toolbox Picture Sequence Memory Test (PSMT)

The PSMT was collected as a traditional measure of episodic memory^47^. Participants were presented with a sequence of cartoon pictures under the theme of “playing at the park” that were presented in a fixed order on an iPad and simultaneously described verbally. Then, participants were instructed to reproduce the sequence three times. Uncorrected standard scores were used to measure performance.

#### Delis-Kaplan Executive Function System (DKEFS) Trail Making Test

The DKEFS Trail Making Test was administered to assess processing speed and cognitive flexibility^48^. In condition A (number sequencing), participants connected numbered circles in ascending order as quickly as possible, providing a measure of psychomotor speed with minimal executive demand. In condition B (number-letter switching), participants alternated between numbered and lettered circles (1–A–2–B–3–C…), indexing set-shifting and cognitive flexibility above and beyond basic motor speed. Completion time (in milliseconds) served as the primary outcome for each condition.

#### Neighborhood Errand Task (NET)

The NET was adapted from the dual-solution paradigm^25^ and constructed in OpenMaze, an open-source toolbox for developing highly customized virtual navigation experiments with cutting-edge graphics and physics engines using Unity^49^. To better approximate the wayfinding approach participants use while navigating through a neighborhood with signs of disorder, the NET virtual environment was rendered to depict a neighborhood with physical indicators of violence, crimes, and social disorder (e.g., dilapidated buildings, excessive litter, abandoned cars; Fig. 1**;** video demo here)https://www.dropbox.com/scl/fi/nlv9qny6g0tkp2ynd5x72/DEMO.mov?rlkey=cp7t10wiqi9mlgfndkscewoh1&dl=0.

Participants were told they were playing a computer game about running errands in a neighborhood. The game consisted of a learning and a testing phase. During learning, participants were taught to use the arrow keys to navigate forward (up), back (down), and turn (left and right, respectively) and given the opportunity to practice navigating to targets (i.e., colored cubes) in a practice virtual environment. Next, more information about the errands and neighborhood were provided. Specifically, participants were told they would run errands to three places (the library, supermarket, and basketball court) and that all errands would happen on different days. Further, participants were told that they might start at a place they’ve seen before and could follow a route they would learn (i.e., the learned route) or that they could start somewhere new and take whatever route they prefer to run the errand. Participants were also told that the violent crime rate in the neighborhood was 10-times the national average, so they would want to run each errand as quickly and safely as possible. Next, as much day-to-day wayfinding is aided by maps^50^, participants were given two minutes to study a simplified overhead map of the neighborhood showing the location of targets at the three places. Then, participants learned the virtual neighborhood by being guided across the learned route indicated with blue arrows, which led them past each of the three places. Participants were guided across the learned route by the experimenter once in each direction and then instructed to follow the learned route in each direction by themselves. Finally, participants completed three practice trials to run an errand to each place for a total of seven learning trials.

During testing, participants completed 18 randomly ordered retrieval trials where they were instructed to go to one of the three places (six trials to each place) with 40 s to navigate. A 2 s crosshair preceded all trials to alert participants to a subsequent 1 s instruction screen indicating the target place for the upcoming trial (e.g., “Go to the library”). Nine trials were “shortcut” trials, where a novel shortcut offered a physically shorter path to the target place than the learned route. The remaining trials were learned route trials, where all novel paths were equivalent or physically longer than the learned route. To increase the sense of neighborhood disorder, 5 surprise startle trials were interspersed across the 18 retrieval trials. The surprise startle trials were triggered by crossing an invisible barrier during wayfinding and featured a sudden white-noise burst paired with a red screen stating, “oh no–you were robbed!” These trials were designed to simulate the kind of sudden, negative disruption one might encounter in a disordered environment — contributing to a broader state of discomfort, unease, and uncertainty that characterizes such contexts. After each surprise startle trial, participants were unable to complete the errand, reflecting the reality that such events disrupt and halt navigation in the real world. Of note, following completion of the NET, participants were asked to rate how much the startle noise bothered them from 1- not at all to 10- a whole lot. Most participants indicated the noise bothered them (61% rated the startle above 5 on the scale, mean = 6.22, SD = 2.79) (see Supplemental Results for change in performance analysis following surprise trials).

Participants were monitored via video throughout the session to ensure task engagement and protocol adherence. To minimize potential distractions and maintain a controlled testing environment, participants were instructed to refrain from eating, using mobile phones, or engaging in any non-task-related activities during the session. These measures were implemented to promote consistent conditions across participants and reduce variability attributable to extraneous factors. Research assistants also recorded participant responses to the startle trials, noting that 80 of the 100 sessions elicited a strong verbal (e.g., yelling profanity, “yelping”) or behavioral (e.g., jumping out of seat) negative reaction.

## Power analysis

The current sample size was determined a priori based on previous research examining individual differences in wayfinding strategies^21,25,51–53^ and simulation-based power analyses conducted using the InteractionPoweR package in R^54^. Power analyses indicated that a sample size of n = 100 would provide sufficient power (i.e., > 80%) for all planned analyses, at an average > 91%, across 10,000 simulations (Supplemental Fig. 2), to detect a medium effect for the interaction between neighborhood disorder and wayfinding.

**Fig. 2:**
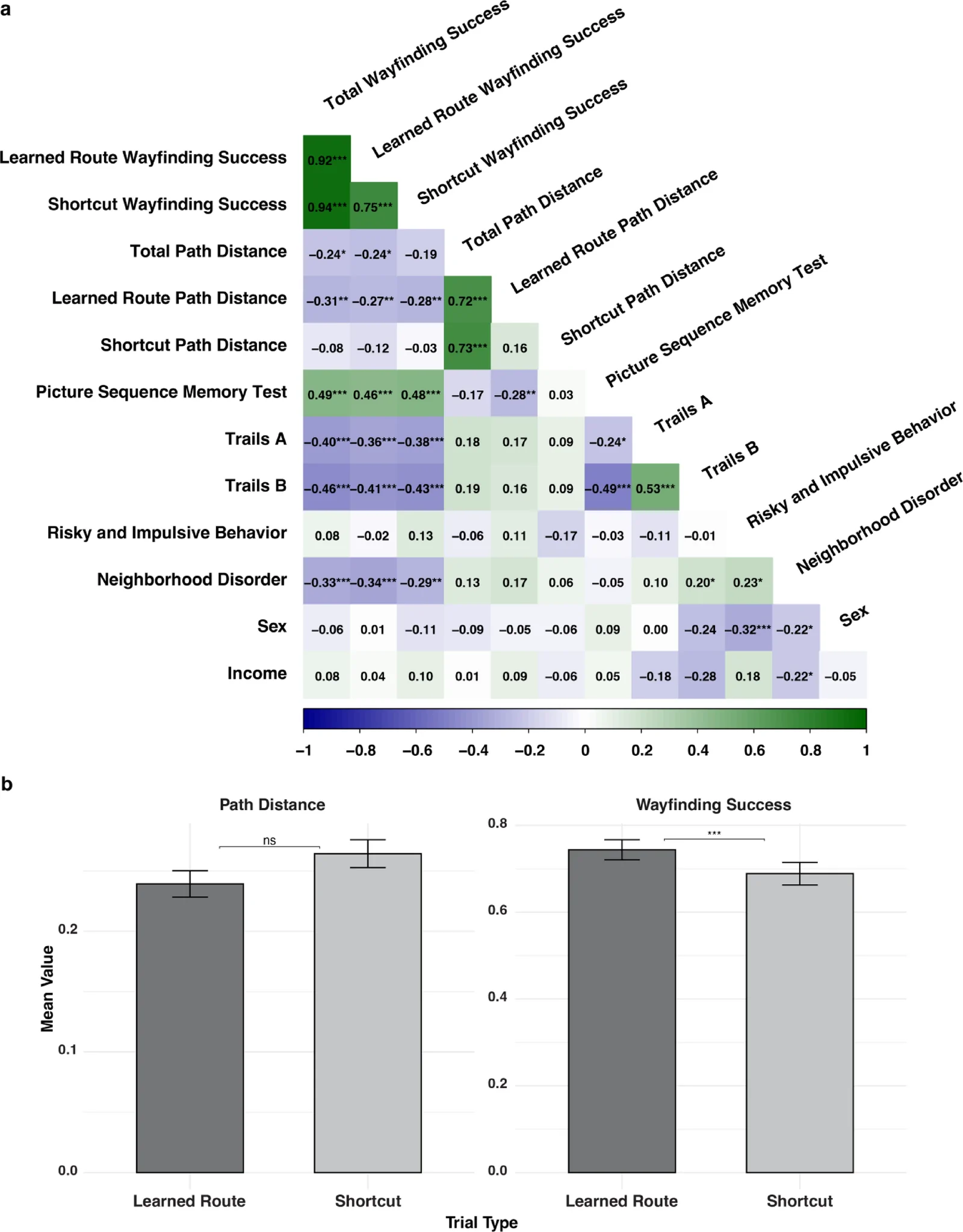
Task performance. (**a**) Spearman correlations between study variables and participant characteristics. (**b**) Bar plots depict means and standard errors for task performance variables.

## Analytic approach

Trial-level behavior data were extracted to assess the NET performance across two measures: wayfinding success and path distance. Wayfinding success was defined as the percentage of trials that were successful in the time allowed. Path distance was calculated by extracting all participant position coordinates (output every 100 ms) and summing the Euclidean distance between all successive coordinate pairs. Because the absolute units from the NET virtual environment were arbitrary, min–max scaling was applied to standardize path distance values to a 0–1 range.

To assess the validity of the NET, we examined associations with the PSMT and Trail Making Test, respectively, compared to task performance measures across learned route and shortcut trials. Given prior work suggesting episodic memory processes support flexible navigation and wayfinding^13–15^, we expected greater PSMT performance to be associated with greater wayfinding success and shorter path distance. We also predicted better performance on learned route trials relative to shortcut trials, which require greater flexible responding and retrieval of episodic information. Given prior work suggesting that processing speed and cognitive flexibility support navigation and wayfinding^55,56^, we expected better Trail Making Test performance (shorter completion time) to be associated with greater wayfinding success. However, we did not expect redundancy across measures given fundamental differences in task design.

Next, we sought to understand whether wayfinding performance during the NET related to participants’ experiences in the real world. First, we examined the influence of perceived neighborhood disorder on wayfinding performance using regression models. Analyses were conducted across all trials as well as separately for learned route and shortcut trials. Next, we tested our a priori hypothesis of wayfinding moderating the relationship between neighborhood disorder and real-world risky behavior. Hierarchical models were used to assess the main effects of perceived neighborhood disorder, wayfinding performance, and their interaction on real-world risky behavior. Johnson-Neyman and simple slopes analyses were used to examine the region of significance for the interaction terms. For brevity, we report significant results from the exploratory moderation analysis (see Supplementary Material for comprehensive results). For all analyses, nonparametric bootstrapping with 1000 resamples was used to provide robust estimates of model parameters and confidence intervals. In addition, effect sizes were examined to assess the magnitude of effects.

## Results

### Basic task effects

We examined the convergent validity between the NET performance and previously validated measures of cognitive functioning. First, we assessed associations with episodic memory processing using the Picture Sequence Memory Test (PSMT). There was a significant positive association between wayfinding success and PSMT scores (ρ_total wayfinding success_ = 0.49 , *p* < 0.001; ρ_learned route wayfinding success_ = 0.46 , *p* < 0.001; ρ_shortcut wayfinding success_ = 0.48 , *p* < 0.001; Fig. 2a). The association between path distance and PSMT scores was significant on learned route trials (ρ_learned route path distance_ = −0.28 , *p* < 0.001), but not on shortcut trials or across all trials (ρ_total path distance_ = −0.17, *p* = 0.11; ρ_shortcut path distance_ = 0.06 , *p* = 0.73).

Then, we examined relationships between the NET performance and the Trail Making Test, a measure of broader cognitive functioning, processing speed, and psychomotor coordination. There was a significant negative association between wayfinding success and Trail Making Test performance on Part A (ρ_total wayfinding success_ = −0.40 , *p* < 0.001; ρ_learned route wayfinding success_ = −0.36 , *p* < 0.001; ρ_shortcut wayfinding success_ = −0.48 , *p* < 0.001; Fig. 2a) and Part B (ρ_total wayfinding success_ = −0.46 , *p* < 0.001; ρ_learned route wayfinding success_ = -0.41, *p* < 0.001; ρ_shortcut wayfinding success_ = −0.43 , *p* < 0.001; Fig. 2a). There were no significant associations between path distance and Trail Making Test performance (Part A: ρ_total wayfinding success_ = 0.18 , *p* = 0.07; ρ_learned route wayfinding success_ = 0.17 , *p* = 0.09; ρ_shortcut wayfinding success_ = 0.09 , *p* = 0.40; Part B: ρ_total wayfinding success_ = 0.19 , *p* = 0.06; ρ_learned route wayfinding success_ = 0.11 , *p* = 0.09; ρ_shortcut wayfinding success_ = 0.09 , *p* = 0.36).

Finally, we assessed whether the NET performance differed between learned route and shortcut trials. Consistent with previous work, wayfinding success was significantly greater on learned route (mean = 0.74 (SD = 0.23)) relative to shortcut (mean = 0.68 (SD = 0.26)) trials [B(SE) = 0.06 (0.02), β = 0.22, *p* < 0.001; Fig. 2b]. Though overall path distance was shorter on learned route (mean = 0.24 (SD = 0.11)) versus shortcut (mean = 0.26 (SD = 0.12)) trials, the difference was not significant (B(SE) = −0.02 (0.02), β = −0.20, *p* = 0.14). See Supplemental Results for checks of task engagement.

## Perceived neighborhood disorder was associated with lower wayfinding success

We examined the influence of perceived neighborhood disorder on the NET performance using linear models with bootstrapped estimates. Specifically, we examined associations between neighborhood disorder and wayfinding success and path distance across all trials, as well as separately for learned route and shortcut trials.

**Fig. 3:**
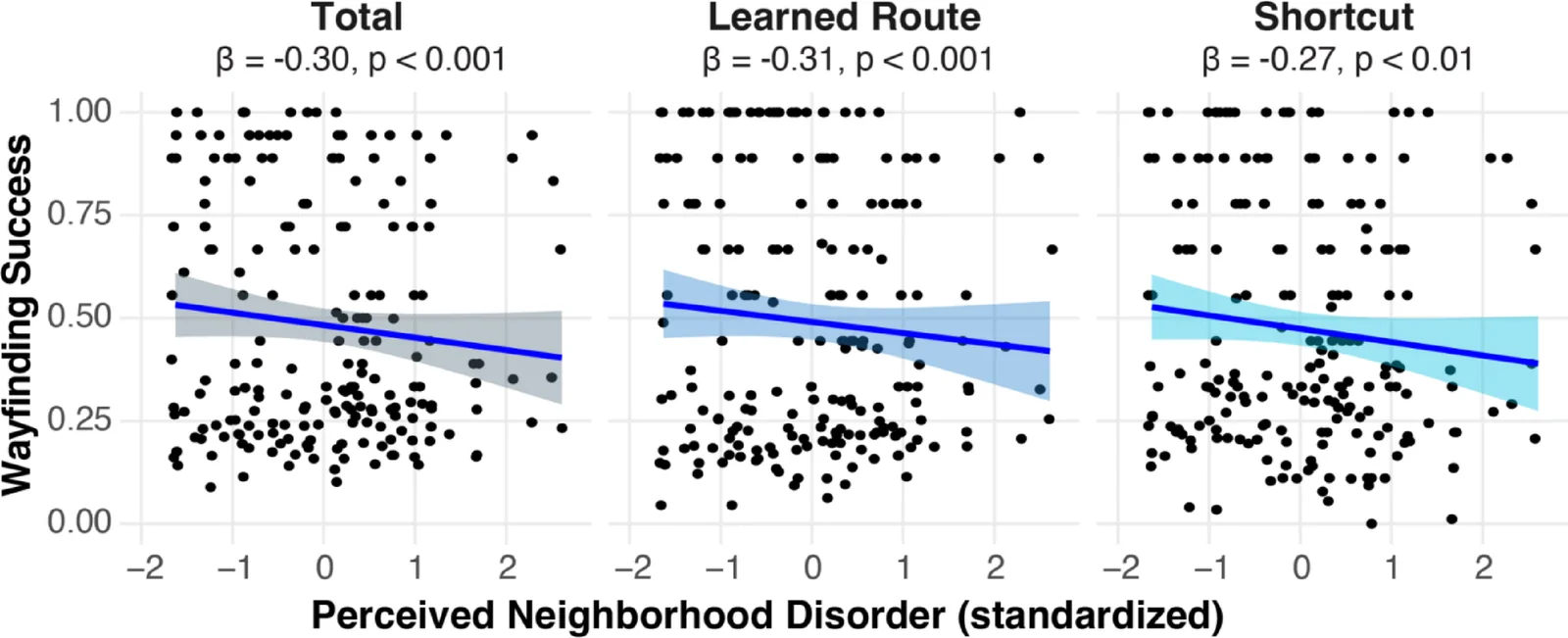
Perceived neighborhood disorder was related to lower wayfinding success. Scatter plots show the distribution of wayfinding success by perceived neighborhood disorder. Error bands represent 95% confidence intervals.

Consistent with our hypotheses, greater perceived neighborhood disorder was significantly related to lower wayfinding success across all trials (B(SE) = −0.07 (0.02), 95% CI = −0.12, −0.03, β = −0.30, *p* < 0.001; Fig. 3). The negative association also was significant when examining performance separately for learned route (B(SE) = −0.07 (0.02), 95% CI = −0.12, −0.03, β = −0.31, *p* < 0.001) and shortcut (B(SE) = −0.07 (0.03), 95% CI = −0.12, −0.02, β = −0.27, *p* < 0.01) trials, suggesting that participants perceiving higher neighborhood disorder had more difficulty with memory and information processing in the NET neighborhood environment compared to those perceiving lower neighborhood disorder.

In contrast, perceived neighborhood disorder was not significantly associated with path distance across all trials (B(SE) = 0.01 (0.01), 95% CI = −0.01, 0.03, β = 0.13, *p* = 0.14) or when examining learned route (B(SE) = 0.02 (0.01), 95% CI = -0.00, -0.004, β = 0.16, *p* = 0.064) and shortcut (B(SE) = 0.01 (0.01), 95% CI = −0.02, 0.03, β = 0.05, *p* = 0.59) trials separately.

### Less efficient wayfinding amplified the association between perceived neighborhood disorder and risky behavior

We conducted a moderation analysis to examine whether wayfinding in a virtual neighborhood with unpredictable events moderated the relationship between perceived neighborhood disorder and real-world risky behavior. For brevity, we report the primary findings from the exploratory analysis (see Supplemental Table 2 for additional results).

**Fig. 4:**
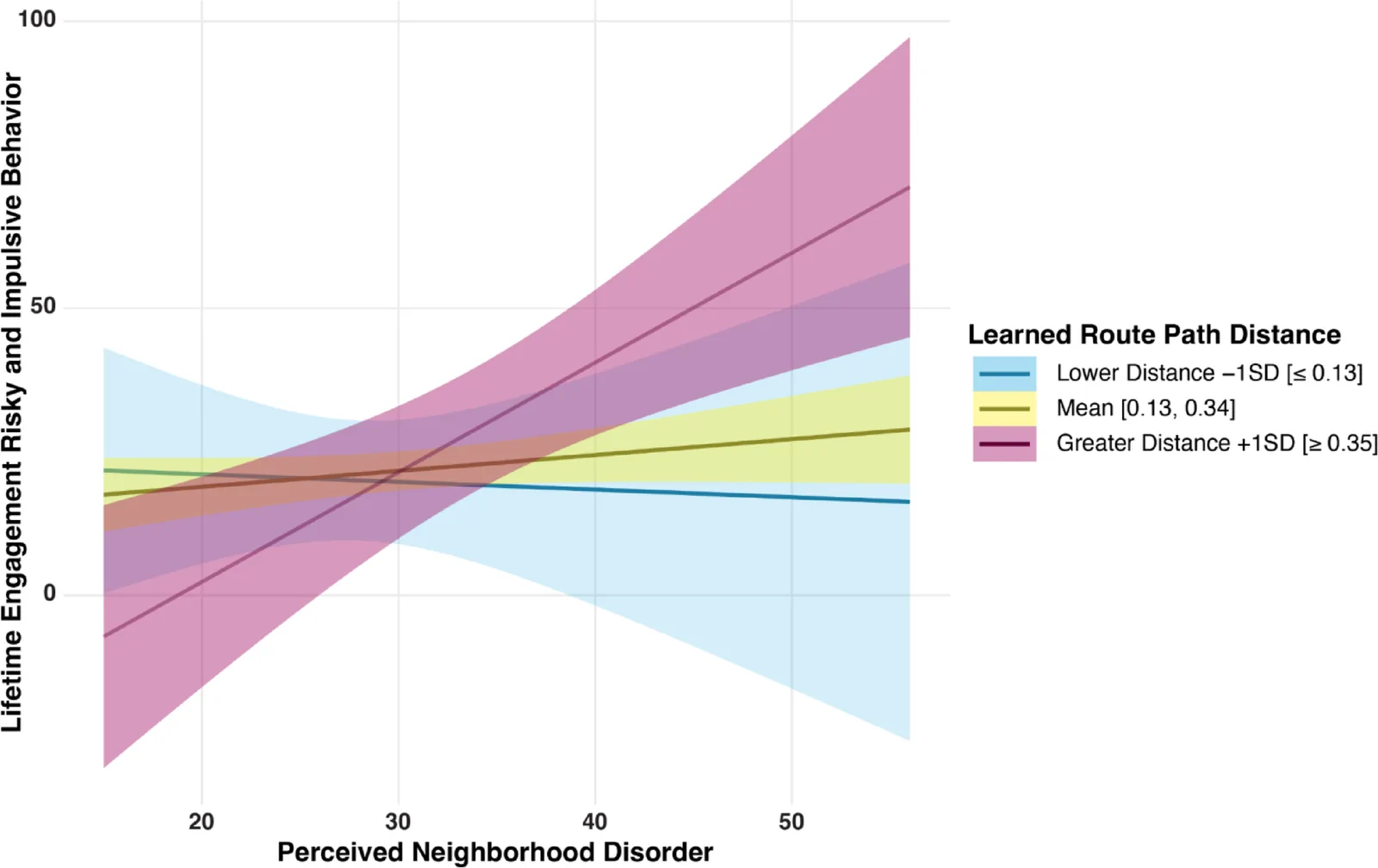
Path distance during learned route trials moderated the association between perceived neighborhood disorder and lifetime engagement in risky and impulsive behavior. Johnson-Neyman analysis demonstrated that the interaction term was only significant for learned route path distance values outside the interval (− 4.27, 0.22) [range of observed learned route path distance values = (0.05, 0.68), mean (SD) = 0.24 (0.11)].

The omnibus model test was significant (F(1, 96) = 4.68, *p* = 0.03) indicating that the interaction between neighborhood disorder and path distance during learned route trials improved model fit. The main effects of neighborhood disorder (B(SE) = − 5.67 (4.96), 95% CI = −13.79—4.22, β = 0.20, *p* = 0.26) and learned route path distance (B(SE) = 15.42 (16.96), 95% CI = −10.04, 51.67, β = 0.09, *p* = 0.37) were not significant. The interaction between perceived neighborhood disorder and path distance on learned route trials was significantly related to real-world risky behavior (B(SE) = 39.93 (18.45), 95% CI = 1.48, 71.25, β = 0.23, *p* = 0.03) such that greater perceived neighborhood disorder was related to increased real-world risky and impulsive behavior among participants who traversed a greater path distance on learned route trials (Fig. 4).

#### Sensitivity results

Additional analyses were conducted to examine the sensitivity of the results described above to the inclusion of covariates. Specifically, we included individual income, PSMT and Trail Making Test performance, and age as additional predictors in the models described above. Replicating the primary results, there was a significant negative relationship between perceived neighborhood disorder and wayfinding success after accounting for variance due to individual income, PSMT and Trail Making Test performance, and age across all trials (B(SE) = − 0.04 (0.02), 95% CI = − 0.08, − 0.00, β = − 0.10, *p* = 0.04) and for the learned route trials separately (B(SE) = − 0.05 (0.02), 95% CI = −0.09, − 0.01, β = − 0.20, *p* = 0.02). After controlling for all covariates, wayfinding success on the shortcut trials was not significant (B(SE) = − 0.04 (0.02), 95% CI = − 0.08, 0.01, β = − 0.14, *p* = 0.10). In addition, path distance on learned route trials significantly moderated the relationship between perceived neighborhood disorder and engagement in risky and impulsive behavior with the inclusion of individual income, PSMT and Trail Making Test performance, and age as covariates (B(SE) = 38.04 (17.70), 95% CI = 2.87, 73.21, β = 0.22, *p* = 0.03).

## Discussion

The present study introduced the Neighborhood Errand Task (NET), a novel virtual navigation paradigm designed to examine how lived experience of neighborhood disorder shapes cognitive functioning in an ecologically valid context. Departing from the tradition of abstract laboratory tasks, the NET embedded wayfinding within a virtual neighborhood rendered with realistic indicators of physical and social disorder (e.g., dilapidated buildings, litter, abandoned cars, and surprise “robbery” events) to more closely approximate the demands individuals face in their daily environments. We found that individuals reporting higher perceptions of neighborhood disorder had more difficulty navigating to goal locations in a virtual neighborhood environment, suggesting individual differences in perceived disorder are meaningfully linked to the cognitive processes that support goal-directed behavior in real-world contexts. Further, among participants who demonstrated less efficient navigation, the association between perceptions of neighborhood disorder and real-world risky and impulsive behavior was amplified. These results suggest that understanding variability in memory and information processing under conditions approximating everyday challenges may be an important step for clarifying how interdependent cognitive processes adapt to individuals’ broader social environments to guide real-world behavior.

A key innovation of the current study was the development of the NET, which captures wayfinding in a virtual neighborhood marked with disorder. In contrast to traditional cognitive tasks, which often focus on isolating cognitive processing under decontextualized and abstracted conditions^12^, the NET simulates memory and information processing under time constraints and unpredictable threats in a context that mirrors the lived environments of individuals exposed to neighborhood disorder. Critically, the NET performance demonstrated convergent validity^57^ with established measures of episodic memory^47^ and processing speed and cognitive flexibility^48^, consistent with prior accounts of wayfinding as a complex, multi-process behavior requiring encoding and retrieval, planning, sustained attention, and flexible responding^19,20^. At the same time, the magnitude of these associations indicated only partial overlap, suggesting the NET captures distinct variance not fully accounted for by traditional decontextualized tasks. Further supporting its validity, wayfinding success was significantly higher on learned route than shortcut trials, replicating prior findings that shortcut use places greater demands on flexible environmental updating and retrieval^25,58^. Together, these findings establish the NET as a tool for assessing cognition in a realistic, adverse environmental context.

Leveraging the NET, results from the current study offer insights into how neighborhood experiences may shape goal-directed behavior. Individuals who reported higher perceptions of neighborhood disorder showed reduced wayfinding success across learned route and shortcut trials, suggesting disruption in memory and information processing that underlies navigation toward a goal. Notably, perceived neighborhood disorder was not associated with path distance during the NET, which may indicate that performance decrements reflected failures to reach destinations within the allotted time rather than aimless or disorganized exploration^59^. These results extend prior work demonstrating that perceptions of disorder^7–9^ and exposure to unpredictable or threatening environments can negatively impact memory and broader cognitive functioning^6,60–62^. Further, results build on laboratory findings showing that induced stress increases reliance on automatic, habit-based responding during wayfinding at the expense of flexible retrieval^23,53,63^. Such a shift toward automatic responding in the context of neighborhood disorder may be functionally adaptive. For example, exposure to neighborhood violence has been associated with heightened hypervigilance for environmental threats^64,65^ and directing attentional resources toward safety monitoring may preserve survival-relevant functioning even as it constrains flexible cognition more broadly^11^. These findings suggest that people who perceive higher levels of disorder in their environments may cognitively adapt to promote automatic strategies that prioritize safety, potentially at the cost of flexible cognitive functioning.

Performance on the NET also was related to real-world behavior. Results from the moderation analysis revealed that inefficient navigation (i.e., longer path distance on learned route trials) amplified the association between perceived neighborhood disorder and risky and impulsive behavior. This finding is consistent with previous research linking neighborhood disorder and memory impairment with behavioral dysregulation^26–28,30^, and suggests that perceptions of neighborhood disorder and navigation inefficiency may interact to increase vulnerability to potentially risky or impulsive behavior. One potential explanation for this relationship could be that navigation inefficiency may decrease individuals’ perceived ability to stay safe in their neighborhood, a construct called “street efficacy”^66^, by increasing exposure to distressing or traumatic experiences related to neighborhood disorder (e.g., being victimized or witnessing violence or crime). In turn, individuals may resort to compensatory risky or impulsive coping strategies such as engaging in aggressive behavior or crime (e.g., carrying weapons, gang involvement) to ward off threats^67,68^ or substance use or other health-risk behaviors to attempt to reduce trauma-related symptoms^69^.

Several limitations warrant discussion. First, the correlational design precludes conclusions about temporal order or causal mechanism. It remains unknown whether navigational inefficiency prospectively predicts risky and impulsive behavior or whether wayfinding serves as one mechanism by which environmental adversity translates into behavioral dysregulation. Relatedly, the absence of a neutral control condition in the NET also limits the specificity of conclusions that can be drawn. Without a low-disorder comparison, it is difficult to determine whether observed effects reflect responses to disorder cues specifically or more general factors such as individual differences in navigation ability or responses to an interrupted, time-pressured task. Although embedding participants in a realistic disordered environment was central to the ecological aims of the study, future work incorporating a matched low-disorder condition would strengthen claims about the unique contribution of disorder cues. Second, although neighborhood disorder was associated with wayfinding success, it was not associated with path distance, leaving the role of navigation strategy partially unclear. The surprise startle trials, while effective at inducing a sense of disorder and unpredictability, may have disrupted path distance as a reliable index of strategy, introducing noise that obscured potential shifts toward habit-based responding^24^. The absence of physiological measures further limits conclusions about whether participants’ reactions to the startle stimuli reflected genuine stress responses. Future research incorporating continuous physiological recording alongside behavioral measures would allow for a more precise characterization of how unpredictable environmental events influence navigation strategy and the affective systems that modulate it. Third, wayfinding is inherently multidetermined, involving the coordination of sensory, motor, spatial memory, planning, and attentional processes^70^. The NET was designed to prioritize representativeness and ecological generalizability over process isolation^71,72^, and as such is better positioned to capture the complexity of everyday navigation than to decompose performance into discrete cognitive components. Identifying which specific processes account for the observed associations between neighborhood disorder and wayfinding success is a valuable next step that will require process-targeted paradigms used alongside the NET. Fourth, participants were recruited from a high-crime urban area to ensure the sample had meaningful lived experience of neighborhood disorder. This strategy was intentional, but limits conclusions about whether the observed associations hold among individuals living in different environments, with little disorder exposure, or remain consistent across the full range of neighborhood conditions. Future work recruiting across more diverse neighborhood contexts would clarify the boundaries of these relationships and whether the reported effects vary as a function of disorder exposure history. Finally, the NET, while ecologically grounded, necessarily simplifies the navigational experience. The virtual neighborhood lacked social interactions, embodied physical effort, and the genuine consequences—safety, time, fatigue—that shape real-world route decisions. Future research should examine the extent to which current findings generalize to immersive virtual reality environments^73^ and, ultimately, to navigation in the real world.

Altogether, the NET offers a new avenue for studying how everyday neighborhood experiences shape cognition and behavior. Results demonstrated that greater perceived neighborhood disorder was associated with disrupted wayfinding, reflecting broad difficulties in the memory and information processing demands that support goal-directed behavior in realistic, adverse environments. Further, navigational inefficiency emerged as a potential vulnerability factor, amplifying the relationship between perceived neighborhood disorder and real-world risky and impulsive behavior. By embedding cognition within an ecologically grounded context, the NET moves beyond isolated laboratory measures to capture how cognitive systems interact dynamically with individuals’ lived environments. This approach has the potential to advance theory on the behavioral consequences of environmental adversity and to inform intervention efforts targeting the pathways through which neighborhood disorder shapes everyday functioning.

## Supplementary Information

Below is the link to the electronic supplementary material.

Supplementary Material 1

## Data Availability

The datasets generated during and/or analyzed during the current study are available from the corresponding author on reasonable request. In addition, the NET standalone application and task code is available for download via https://www.dropbox.com/scl/fo/clq6upma4im80krigl0zq/AJ2mMtsENmimfbe8Wi24_Mg?rlkey=w9lqv30ud0wdpjb4oiov7hypm&dl=0.
