# Supplementary material for: Neighborhood disorder impacts cognitive processing during navigation on a novel virtual reality paradigm
Source: Sci Rep. 2026 Jun 9;16:26216. doi: 10.1038/s41598-026-56852-4 (PMC13493969; doi:10.1038/s41598-026-56852-4)
Supplement: Supplementary file 1 [file 41598_2026_56852_MOESM1_ESM.docx]

**Neighborhood disorder impacts cognitive processing
during navigation on a novel virtual reality paradigm**

**Supplementary Material**

**Supplemental Materials and Methods**

**Supplemental Figure 1**. Histogram of Perceived Neighborhood Disorder scores in the present sample. The range of possible scores is 15 to 60.

**Supplemental Figure 2**. Results from the a priori simulation-based power analyses showing power as a function of sample size. The blue line depicts target power at 80%.

**Supplemental Table 1.** Participant characteristics and key task variables

| ***n*** | 100 |
| --- | --- |
| **Age (mean (SD))** | 32.58 (9.58) |
| **Female (%)** | 0.41 (0.49) |
| **Race/Ethnicity (%)** |  |
| AIAN | 1 (1.0) |
| Asian | 13 (13.0) |
| Black/African American | 31 (31.0) |
| Hispanic or Latinx | 7 (7.0) |
| MENA | 1 (1.0) |
| Mixed | 11 (11.0) |
| Other | 6 (6.0) |
| White | 30 (30.0) |
| **Individual Income (%)** |  |
| < $5,000 | 19 (19.0) |
| $5,000 through $11,999 | 12 (12.0) |
| $12,000 through $15,999 | 7 (7.0) |
| $16,000 through $24,999 | 12 (12.0) |
| $25,000 through $34,999 | 10 (10.0) |
| $35,000 through $49,999 | 14 (14.0) |
| $50,000 through $74,999 | 11 (11.0) |
| $75,000 through $99,999 | 12 (12.0) |
| $100,000 and greater | 3 (3.0) |
| **Perceived Neighborhood Disorder** **(mean (SD))** | 30.75 (9.67) |
| **Lifetime RISQ** **(mean (SD))** | 22.42 (19.11) |
| **Wayfinding Success** |  |
| Total (mean (SD)) | 0.72 (0.23) |
| Learned Route (mean (SD)) | 0.74 (0.23) |
| Shortcut (mean (SD)) | 0.69 (0.26) |
| **Path Distance** |  |
| Total (mean (SD)) | 0.25 (0.08) |
| Learned Route (mean (SD)) | 0.24 (0.11) |
| Shortcut (mean (SD)) | 0.26 (0.12) |

**Supplemental Results**

**Basic Task Effects: Task Engagement**

As a check of task engagement across the session, we examined reaction time throughout the session. There was no mean difference in reaction time between beginning and end trials (t = 1.50, p-value = 0.14), and this did not vary by PND (B(SE)=.0002 (.004), p=.97). We also found that reaction time decreased across the task, indicating an anticipated learning effect (B(SE)=-0.13 (.04), p=.001). This overall learning effect, importantly, did not vary by PND (B(SE)=.0001 (.004), p=.971). Therefore, participants appeared behaviorally engaged throughout the task and this did not seem to systematically vary by our primary individual difference (PND).

**Basic Task Effects: Change in Performance Following Startle Trials**

We analyzed potential change in performance immediately after the startle trials (i.e., comparing the odds of improved versus worsened performance after the “robbery” events when there was a change in performance). Overall, accuracy changed following startle trials (OR = 0.84), corresponding to a -2.8 percentage-point change in accuracy relative to before startle trials. On average, when before/after performance was discordant (reflecting a change in performance before/after startle), post-startle performance worsened most of the time (54.3%).

**Supplemental Table 2.** Ancillary results from the exploratory moderation analysis

| **Total Path Distance** | | | | |
| --- | --- | --- | --- | --- |
| *Predictors* | *Estimates (SE)* | *β* | *95% CI* | *p* |
| PND | -0.05 (6.63) | 0.25 | -13.21, 13.11 | 0.994 |
| Total Path Distance | -22.74 (24.14) | -0.09 | -70.67, 25.18 | 0.349 |
| PND × Total Path Distance | 19.20 (25.05) | 0.08 | -30.52, 68.92 | 0.445 |
| **Shortcut Path Distance** | | | | |
| *Predictors* | *Estimates (SE)* | *β* | *95% CI* | *p* |
| PND | 8.07 (5.00) | 0.24 | -1.85, 18.00 | 0.110 |
| Shortcut Path Distance | -33.80 (16.54) | -0.20 | -66.63, -0.97 | **0.044** |
| PND x Shortcut Path Distance | -13.15 (18.49) | -0.08 | -49.85, 23.55 | 0.479 |
| **Total Wayfinding Success** | | | | |
| *Predictors* | *Estimates (SE)* | *β* | *95% CI* | *p* |
| PND | 10.14 (7.55) | 0.28 | -4.86, 25.14 | 0.183 |
| Total Wayfinding Success | 9.80 (8.73) | 0.12 | -7.54, 27.14 | 0.265 |
| PND × Total Wayfinding Success | -6.57 (9.64) | -0.08 | -25.70, 12.55 | 0.497 |
| **Shortcut Wayfinding Success** | | | | |
| *Predictors* | *Estimates (SE)* | *β* | *95% CI* | *p* |
| PND | 7.07 (6.22) | 0.29 | -4.31, 26.98 | 0.154 |
| Shortcut Wayfinding Success | 11.93 (7.50) | 0.16 | -13.84, 21.42 | 0.670 |
| PND x Shortcut Wayfinding Success | -2.31 (8.14) | -0.03 | -27.82, 10.86 | 0.386 |
| **Learned Route Wayfinding Success** | | | | |
| *Predictors* | *Estimates (SE)* | *β* | *95% CI* | *p* |
| PND | 11.33 (7.88) | 0.26 | -4.31, 26.98 | 0.154 |
| Learned Route Wayfinding Success | 3.79 (8.88) | 0.05 | -13.84, 21.42 | 0.670 |
| PND × Learned Route Wayfinding Success | -8.48 (9.74) | -0.10 | -27.82, 10.86 | 0.386 |

*Note:* PND = Perceived Neighborhood Disorder
